# Fungal Mobilization of Selenium in the Presence of Hausmannite and Ferric Oxyhydroxides

**DOI:** 10.3390/jof7100810

**Published:** 2021-09-28

**Authors:** Bence Farkas, Hana Vojtková, Marek Bujdoš, Marek Kolenčík, Martin Šebesta, Michaela Matulová, Eva Duborská, Martin Danko, Hyunjung Kim, Kateřina Kučová, Zuzana Kisová, Peter Matúš, Martin Urík

**Affiliations:** 1 Institute of Laboratory Research on Geomaterials, Faculty of Natural Sciences, Comenius University in Bratislava, Mlynská Dolina, Ilkovičova 6, 842 15 Bratislava, Slovakia; farkas62@uniba.sk (B.F.); marek.bujdos@uniba.sk (M.B.); martin.sebesta@uniba.sk (M.Š.); michaela.matulova@uniba.sk (M.M.); eva.duborska@uniba.sk (E.D.); peter.matus@uniba.sk (P.M.); 2 Department of Environmental Engineering, Faculty of Mining and Geology, VŠB–Technical University of Ostrava, 17. Listopadu 15/2172, 708 00 Ostrava, Czech Republic; hana.vojtkova@vsb.cz (H.V.); kucova.katerina@gmail.com (K.K.); 3 Institute of Agronomic Sciences, Faculty of Agrobiology and Food Resources, Slovak University of Agriculture in Nitra, Tr. A. Hlinku 2, 949 76 Nitra, Slovakia; marekkolencik@gmail.com; 4 Polymer Institute, Slovak Academy of Sciences, Dúbravská Cesta 9, 845 41 Bratislava, Slovakia; upoldan@savba.sk; 5 Department of Mineral Resources and Energy Engineering, Jeonbuk National University, Jeonju 54896, Jeonbuk, Korea; kshjkim@jbnu.ac.kr; 6 Department of Environment and Energy, Jeonbuk National University, Jeonju 54896, Jeonbuk, Korea; 7 Institute of Molecular Biology, Slovak Academy of Sciences, Dúbravská Cesta 21, 845 51 Bratislava, Slovakia; zuzana.kisova@savba.sk

**Keywords:** bioleaching, fungi, iron, manganese, selenate

## Abstract

Bioleaching of mineral phases plays a crucial role in the mobility and availability of various elements, including selenium. Therefore, the leachability of selenium associated with the surfaces of ferric and manganese oxides and oxyhydroxides, the prevailing components of natural geochemical barriers, has been studied in the presence of filamentous fungus. Both geoactive phases were exposed to selenate and subsequently to growing fungus *Aspergillus niger* for three weeks. This common soil fungus has shown exceptional ability to alter the distribution and mobility of selenium in the presence of both solid phases. The fungus initiated the extensive bioextraction of selenium from the surfaces of amorphous ferric oxyhydroxides, while the hausmannite (Mn_3_O_4_) was highly susceptible to biodeterioration in the presence of selenium. This resulted in specific outcomes regarding the selenium, iron, and manganese uptake by fungus and residual selenium concentrations in mineral phases as well. The adverse effects of bioleaching on fungal growth are also discussed.

## 1. Introduction

Iron and manganese oxides and (oxy)hydroxides belong to geochemically active phases that are involved in the trapping of trace elements in soils and sediments [1]. This is instigated via adsorption, coprecipitation, and redox processes that occur on their surfaces [2]. Since the manganese oxides are among the strongest redox-active naturally occurring minerals, and the sorptive properties of ferric precipitates toward both cations and anions are excellent [3,4], the deposition of oxygenated iron and manganese phases significantly affects the speciation and mobility of various essential and potentially toxic elements in the environment, including selenium [5,6].

Selenium is a trace element that plays an important role in human health [7,8]. However, high doses of selenium can be toxic and have various adverse effects on the immune and neuropsychological functions of organisms [9]. Therefore, its elevated concentration in soils and sediments poses a serious environmental threat, and thus, various treatment techniques to deselenify the selenium-contaminated sites have been proposed [10]. However, its concentration in the soils and sediments is usually low [11], and its deficiency in the diet is a widespread problem [12]. Nevertheless, selenium is a widely commercially used material, and it is also generated during the processing of sulfidic ores or excessive agricultural irrigation [13]. Therefore, it can enter the environment in elevated concentrations and cause severe environmental issues, e.g., the contamination of Kesterson Reservoir (CA, USA) via subsurface agricultural drainage water [14].

Although the manganese and ferric oxides’ and (oxy)hydroxides’ surfaces represent excellent natural active sites for selenium immobilization [15,16,17], it was reported that their binding capacities can be significantly altered via interactions with microorganisms [18,19]. In the case of the filamentous fungi, this is due to the exudation of redox-active, chelating, and acidic metabolites that interact with the minerals’ surfaces directly or indirectly while altering their morphology, surface properties, and mineralogy via dissolution and recrystallization [20,21,22]. Furthermore, the fungus can actively disrupt the dynamic equilibrium at the solid phases’ and solution’s interfaces via accumulation and subsequent compartmentalization of the extracted elements [23]. This effectively limits the readsorption of the extracted elements and, thus, enhances the bioextraction efficiency. Alternatively, the accumulated elements can be transformed intracellularly by the fungus into new species whose affinity toward the manganese and ferric oxides’ and (oxy)hydroxides’ surfaces may significantly differ (e.g., the degree of methylation alters the sorption behavior of the transformants) [24].

Since selenium is not considered a global contaminant, there is a lack of scientific effort and focus on studying the fungal effects on selenium mobility in the presence of manganese and ferric oxides and (oxy)hydroxides in comparison to other potentially toxic oxyanions, including chromium, arsenic, and antimony. This inspired us to study the alterations in selenium distribution and mobility in the presence of the common soil fungus *Aspergillus niger*, which is one of the most studied fungal strains regarding the issue of bioleaching of natural and synthetic mineral phases.

## 2. Materials and Methods

### 2.1. Microorganism

Spore suspension for the culture media inoculation was prepared from a 7-day old fungal colony of *Aspergillus niger* CBS 140837 strain. The fungus was isolated from mercury-contaminated soil [25] and is kept in The Mycological Laboratory collection at Slovak Medical University in Bratislava (Bratislava, Slovakia).

### 2.2. Ferric Oxyhydroxides and Hausmannite Synthesis

Ferric oxyhydroxides used in this study were prepared by alkaline (40 g NaOH p.a.; Centralchem, Bratislava, Slovakia) precipitation of FeCl_3_ (54.06 g FeCl_3_.6H_2_O p.a.; Centralchem, Bratislava, Slovakia) in 1 L of deionized water under laboratory conditions. After 12 h stirring at 150 rpm (Unimax 2010; Heidolph, Schwabach, Germany), freshly prepared precipitates were filtered, washed with distilled water, and dried at 80 °C overnight. The synthesized ferric precipitate was stored in a sealed container at room temperature before further characterization and experiments.

Manganese oxides used in this study were prepared by alkaline (40 g NaOH) precipitation of MnSO_4_ (111.5 g MnSO_4_.6H_2_O p.a.; Centralchem, Bratislava, Slovakia) in 1 L of deionized water under laboratory conditions. After 5 h heating under reflux, the freshly prepared precipitate was filtered, washed with distilled water, and dried at 80 °C. The precipitate was then oven-heated at 95 °C for one hour prior to use and analysis.

Samples were characterized by X-ray powder diffraction (XRD) analyses elsewhere [22,23] and were identified as amorphous ferric oxyhydroxides and hausmannite [Mn_3_O_4_].

### 2.3. Bioleaching

Series of 45 mL culture Sabouraud Dextrose Broth Media (HiMedia, Mumbai, India) in 100 mL Erlenmeyer flasks autoclaved at 121 °C for 15 min were supplemented with 0.11 g of amorphous ferric oxyhydroxides or hausmannite. Except for selenium-free control experiments, all treatments were supplemented with 5 mL of 500 mg·L^−1^ selenium (VI) concentration, prepared from Na_2_SeO_4_ p.a. (Fluka-Sigma Aldrich, Saint-Louis, MO, USA). Therefore, the final pulp density of ferric oxyhydroxides and hausmannite was 2.2 g·L^−1^, with the total iron and manganese concentrations of 1.4 g·L^−1^ and 1.6 g·L^−1^, respectively. The final concentration of total selenium in the selenium(VI)-treated media was 50 mg·L^−1^. The suspensions were stirred at 130 rpm for 24 h; one portion was then sampled for the total selenium concentration in both solid phases and medium, and the other portion was subsequently inoculated with fungal spores, followed by the static three-week incubation at 25 °C in the dark.

Thereafter, the fungal biomass was separated from the culture media, washed with distilled water, and dried at 25 °C. The spent culture media were then filtered through a filter membrane with 0.45 μm pore size. Dry biomass weight and pH values (HI1230B; Hanna Instruments, Woonsocket, RI, USA) of each culture filtrate including controls were determined. The residual non-dissolved solid phases were air-dried and digested. The biomass, non-dissolved residue (after digestion), and the spent culture media were analyzed for total selenium, manganese, and iron by inductively coupled plasma–optical emission spectrometry (ICP-OES) or flame atomic absorption spectrometry (F AAS). Control experiments were carried out without either selenium or ferric oxyhydroxides and hausmannite, following the same protocol. All treatments were in triplicate.

### 2.4. Analytics

Culture filtrate, dried biomass, and insoluble residue were analyzed for total selenium content by ICP-OES at line Se I 196.090 nm using an ICP spectrometer Jobin Yvon 70 Plus (Horiba Jobin Yvon, Longjumeau, France) equipped with a concentric nebulizer (Meinhard, Golden, CO, USA) and cyclonic spray chamber; plasma power: 1000 W. The accuracy of the method was checked using certified reference materials BCR-185 (bovine liver) and NCS DC73350 (poplar leaves; China National Analysis Center for Iron and Steel, Beijing, China). The results were in agreement with certified values within their uncertainties. The precision of the method was 5% (relative combined standard uncertainty with coverage factor k = 2).

The total manganese and iron content were analyzed by F AAS at lines Mn 279.5 nm and Fe 248.3 nm using an AAS spectrometer PerkinElmer Model 1100 (Perkin Elmer Instruments, Überlingen, Germany). Deuterium background correction was used for both elements. Calibration solutions were prepared from CertiPUR ICP 1000 mg·L^−1^ single-element standard solutions (Merck, Darmstadt, Germany). The accuracy of the method was checked using certified reference materials NIST SRM 1577c (bovine liver), NCS DC73350 (poplar leaves; China National Analysis Center for Iron and Steel, Beijing, China), and CRM Astasol AN9090MN (aqueous multi-element standard solution; Analytika, Praha, Czech Republic). The results were in agreement with certified values within their uncertainties. The method’s precision was 4% for manganese and 6% for iron (relative combined standard uncertainty with coverage factor k = 2).

## 3. Results

### 3.1. pH of the Culture Media

**Figure 1:**
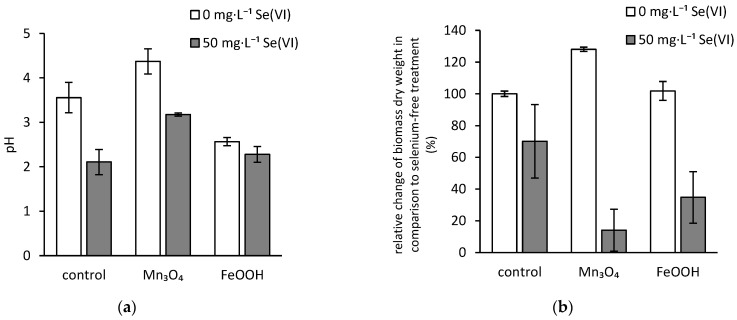
Effects of selenium on (**a**) pH and (**b**) biomass synthesis during the three-week static incubation of hausmannite (Mn_3_O_4_) or amorphous ferric oxyhydroxides (FeOOH) in the presence of filamentous fungus *Aspergillus niger* (control represents the treatment without any solid inorganic phases).

Except for the amorphous ferric oxyhydroxides-treated media, the presence of selenate resulted in a statistically significant decrease of pH at the end of the three-week cultivation (Figure 1a). Since the final average values of pH in selenium-free treatments were 3.6 and 4.4 for the hausmannite-free and hausmannite-treated samples, respectively; we hypothesized that the addition of selenate affected the fungal development and activity to the extent that the exudation of acidic metabolites was enhanced. It resulted in final pH of 2.1 and 3.2 for the hausmannite-free and hausmannite-treated samples, respectively. However, there was no statistically significant difference in the final pH of selenium-treated and selenium-free media supplemented with amorphous ferric oxyhydroxides.

### 3.2. Dry Biomass Weight

The negative effects of selenium on fungal growth were statistically significant in comparison to the control, regardless of the addition of solid inorganic phases (Figure 1b). However, the presence of solid ferric and manganese matrices in the medium enhanced the negative impact of selenate on the fungal development, and the 86% and 65% average growth inhibition were recorded, respectively. This was unexpected since the fungus reacted to manganese in selenium-free media favorably, and the amorphous ferric oxyhydroxides did not have any statistically significant effect on fungal growth.

### 3.3. Bioextraction of Iron, Manganese, and Selenium

**Figure 2:**
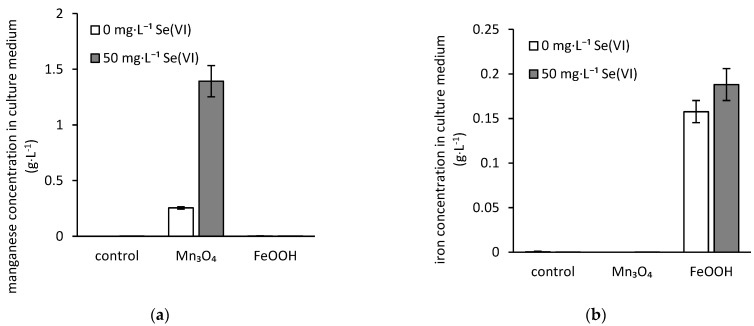
Changes in the concentration of dissolved (**a**) manganese and (**b**) iron in the selenium-free or selenate-treated culture media after the three-week static incubation of hausmannite (Mn_3_O_4_) or amorphous ferric oxyhydroxides (FeOOH) in the presence of fungus *Aspergillus niger* (control represents the treatment without any solid inorganic phases).

Acidification of media by the fungal metabolites led to the significant release of manganese from hausmannite during cultivation (Figure 2a). While 0.26 g·L^−1^ of manganese was recorded in the culture media in the absence of selenium, its presence triggered the manganese release from hausmannite whose dissolution rate was five times higher in comparison to selenium-free treatment. However, there was only a marginal difference in the concentration of iron bioextracted from the amorphous ferric oxyhydroxides in both selenium presence and absence with the average values of 0.19 g·L^−1^ and 0.16 g·L^−1^, respectively (Figure 2b).

**Figure 3:**
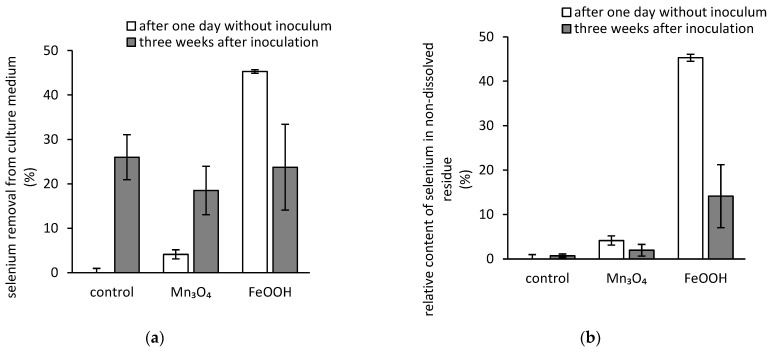
Relative changes of selenium concentrations in (**a**) culture media and (**b**) residual ferric and manganese non-dissolved residues before inoculation and after the three-week static incubation of hausmannite (Mn_3_O_4_) or amorphous ferric oxyhydroxides (FeOOH) in the presence of fungus *Aspergillus niger* and selenium, which was introduced into the medium in the form of selenate (control represents the treatment without any solid inorganic phases; initial concentration of total selenium in culture medium was 50 mg·L^−1^).

There was no statistically significant difference in selenium concentrations recorded in the culture media for any treatment at the end of the three-week cultivation (Figure 3a). The growing fungus was capable of accumulating up to 26% of selenium from media on average after three weeks. However, the initial removal of selenium (after 24 h without inoculum), which was attributed solely to selenium sorption onto the minerals’ surfaces, was approximately 4% and 45% for hausmannite and amorphous ferric oxyhydroxides treated media, respectively. The latter indicated that the biologically induced desorption of selenium from the surfaces of amorphous ferric oxyhydroxides took place during the cultivation, since in the presence of oxyhydroxides the removal capacity decreased from the initial 45% to approximately 24% after three weeks.

The aforementioned amorphous ferric oxyhydroxides’ susceptibility to bioextraction of selenium coincided well with its content decrease in the residual solid ferric phases from approximately 1.2 mg (45% of initial selenium content) at the initial day of inoculation to 0.4 mg (approximately 14% of initial selenium content) after three weeks of incubation (Figure 3b). Selenium content in the hausmannite did also decrease by approximately 50% after three weeks of incubation (Figure 3b). However, since its concentration in media has also decreased during cultivation (Figure 3a), the uptake of selenium by fungal biomass played most likely a more prominent role in this case.

### 3.4. Bioaccumulation of Iron, Manganese, and Selenium

**Figure 4:**
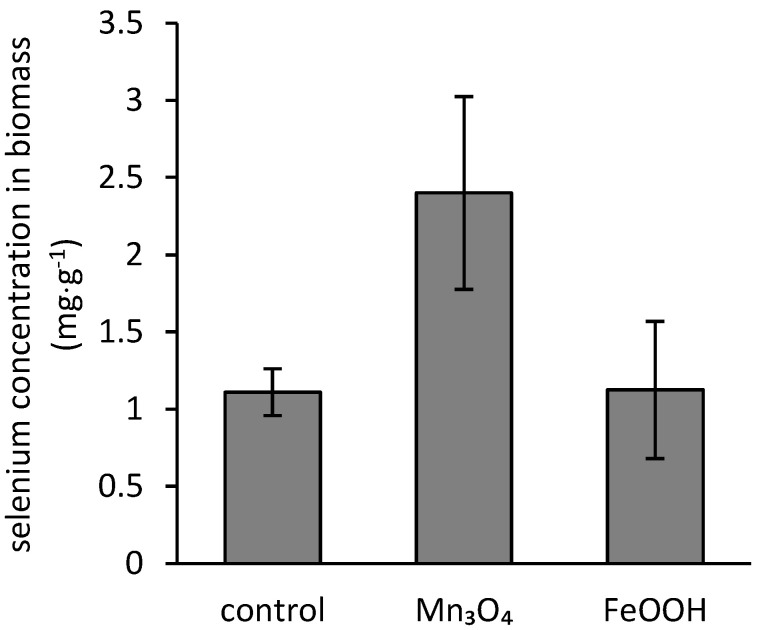
The effects of hausmannite (Mn_3_O_4_) and amorphous ferric oxyhydroxides (FeOOH) on the bioaccumulation of selenium by the biomass of *Aspergillus niger*.

**Figure 5:**
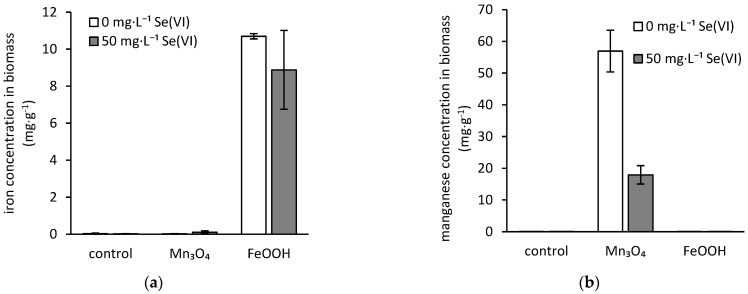
Changes in concentration of (**a**) iron and (**b**) manganese in biomass of *Aspergillus niger* after being cultivated for three weeks on selenium-free or selenate-treated culture media in the presence of hausmannite (Mn_3_O_4_) or amorphous ferric oxyhydroxides (FeOOH) (control represents the treatment without any solid inorganic phases).

During the cultivation, the fungus *A. niger* has shown the ability to bioaccumulate selenium and iron, and manganese as well. While there was no statistically significant difference in comparison of selenium concentration in biomass of the control treatment and biomass incubated in the presence of ferric phases (on average of 1.1 mg·g^−1^), manganese phases statistically significantly increased the amount of bioaccumulated selenium (2.4 mg·g^−1^) (Figure 4). On the other hand, the presence of selenium reduced the manganese accumulation, while the iron concentration in biomass did not undergo a statistically significant change (Figure 5).

## 4. Discussion

We have previously reported that the ferric oxyhydroxides have a great affinity for both dominant oxygenated species of selenium in acidic solutions [26,27]. Here, our results showed that after 24 h the amount of removed selenate by amorphous ferric oxyhydroxides reached 45%. This corresponds to approximately 10.5 mg·g^−1^ sorption capacity, which is similar to other reported values, e.g., the maximum sorption capacities of 15.1 mg·g^−1^ and 22.5 mg·g^−1^ reported for nanoparticles of ferric oxyhydroxides and hydrous ferric oxides impregnated in a polymeric matrix, respectively [28,29]. However, exposure to filamentous fungus *A. niger* significantly decreased the ability of ferric phases to immobilize selenium since its average content in ferric amorphous oxyhydroxides decreased by 70% after cultivation (Figure 3).

The factor that generates the phenomenon of selenium bioextraction is most likely the exudation of iron-chelating metabolites, e.g., fungal siderophores [30] and citrate [31], and extrusion of free protons as well [32]. Osman et al. [33] noted that the *A. niger*’s siderophores (e.g., ferrichrome) are produced in high quantities in media under iron limitation. Therefore, the contribution of siderophores to the biodeterioration of ferric phases is questionable, since the medium used in this study was not iron deficient, and these metabolites were not quantified. Similarly, the citrate also seems to be produced to overcome the iron limitation. However, the surplus of saccharose may facilitate citrate production via overflow metabolism [34]. Furthermore, *A. niger* is an efficient acidifier of its surroundings via the exudation of protons and a well-known producer of other low-molecular-weight organic acids, e.g., oxalate, whose extrusion depends on the ambient pH [35]. It was reported that both low-molecular-weight organic acids and protons contribute to the dissolution of ferric minerals [31,36]. Since our previous research indicated that both citrate and oxalate are produced by the fungal strain used in the presented study [37,38], and the strain is also capable of acidifying the media during cultivation (Figure 1a), we can hypothesize that the aforementioned metabolites and extruded protons deteriorate the matrix of ferric oxyhydroxides or oxides, and the iron ions are then released into the solution [39]. This is highlighted by the recorded concentration of dissolved iron in the culture medium after cultivation that reached 190 mg·L^−1^ (Figure 2b). Thus, the medium acidification via fungal activity (pH ˂ 3; Figure 1a) favored the dissolution of ferric phases and resulted in distortion of equilibrium selenium concentration at amorphous ferric oxyhydroxides’ surfaces. Thus, the bioleaching of amorphous ferric oxyhydroxides should enhance the availability of selenium for the fungus.

The preceding statement is supported by the bioaccumulation data illustrated in Figure 4. It was reported by Urík et al. [40] that the extent of selenium bioaccumulation is affected by the initial concentration of selenate in the medium. Therefore, since the amorphous ferric oxyhydroxides decreased the initial concentration of free selenium in the medium by 45% (Figure 3), we expected that the bioaccumulation capacity would also be lower. However, the concentrations of uptaken selenium after three-week cultivation were not statistically different for the control and the treatment supplemented with amorphous ferric oxyhydroxides (Figure 4). This highlights the environmental relevance of biological deterioration of ferric phases in soils and sediments for nutrients’ bioavailability since the ferric oxides and oxyhydroxides affect the dynamics of various essential elements via sorption and desorption [41]. The general ability of fungal metabolites to enhance the mobility of elements via interactions with mineral surfaces has immense technological implications [42], but it still poses a hazard for the environment in contaminated areas regarding the mobilization of potentially toxic elements [43], including selenium [44].

It was reported that the *A. niger* strain is relatively sensitive to selenate even at a concentration as low as 20 mg·L^−1^ [45]. Therefore, since the initial concentration of selenium in our experiment was higher (50 mg·L^−1^), the growth inhibition of fungus was expected. The toxic effect should have been mitigated by the presence of solid ferric/ferrous iron and manganese oxides and oxyhydroxides [46] that would limit the bioavailable fraction of selenium during initial growth via sorptive interactions or precipitation. However, the presence of iron and manganese solid phases enhanced the adverse effects of selenium on fungal physiology and resulted in synergistic toxic effects (Figure 1b). The abiotically and biologically driven surface interactions are complex and may alter the speciation of selenium [47]. Thus, we suggest that the occurrence of selenate transformants, including selenite that is reportedly more toxic [48], could result in higher sensitivity of fungus to selenium in the presence of amorphous ferric oxyhydroxides and hausmannite as well.

Hausmannite’s effect on selenium mobility in the presence of filamentous fungus is contrasting the behavior of selenate in media treated with amorphous ferric oxyhydroxides. Its affinity toward selenium is significantly lower, and thus, the bioextraction is also less pronounced (Figure 3). Balistrieri and Chao [49] reported that this is due to intrinsic differences in the binding properties of the ferric oxyhydroxides’ and manganese oxides’ surfaces and the manganese oxides exhibited negligible affinity toward selenate even under acidic conditions.

Interestingly, the presence of selenium triggered the bioleaching of hausmannite. Thus, the concentration of free manganese in the medium increased more than five times compared to selenium-free treatment (Figure 2a). Since the fungi are capable of dissolving manganese oxides via both reductive and oxidative pathways via extracellular metabolites [50,51], it is very likely that the presence of selenium prompted extrusion of redox-active metabolites or enzymes responsible for hausmannite dissolution, e.g., exposure of fungus *Beauveria caledonica* to heavy metals led to overexcretion of citric and oxalic acids, with the former reported to actively contribute to the reductive dissolution of manganese oxides in acidic solutions [52].

The exposure to elevated manganese concentration also led to activation of an intrinsic cell mechanism restricting the manganese uptake by fungus, or it intensified its extrusion from the fungal cells (Figure 5b). This is due to the adverse effect of high manganese concentrations on *A. niger* strain, which was reported elsewhere [53]. Still, in the absence of selenium, the beneficial effect of hausmannite on fungal growth was recorded (Figure 1b). Therefore, the threshold for the activation of the protective cell mechanism should lay somewhere between 0.25 and 1.4 mg·L^−1^ concentrations of dissolved manganese in the medium (Figure 2). Nevertheless, the exposure of fungus to elevated concentrations of manganese, in conjunction with the higher amounts of bioaccumulated selenium (Figure 4), resulted in an 86% decrease in fungal growth.

## 5. Conclusions

In summary, the common soil filamentous fungus *A. niger* showed the ability to alter the distribution and mobility of selenium in the presence of both hausmannite and amorphous ferric oxyhydroxides, with the latter having a great affinity toward selenate while still being highly susceptible to selenium desorption via fungal activity. On the other hand, the low sorption capacity of hausmannite resulted in higher exposure of fungus to selenium, which intensified the bioaccumulation of selenium and, thus, the detrimental effect on biomass synthesis. Furthermore, the hausmannite was significantly degraded in the presence of selenium, which triggered hausmannite’s dissolution. The leached manganese further enhanced the harmful effects and resulted in manganese’s restricted uptake by *A. niger* strain. To conclude, our results provided unique insights into the consequences of the ferric and manganese (hydro)oxides’ bioleaching, especially regarding selenium mobility and bioaccumulation.

## Data Availability

Data is contained within the article.
